# Edge-Based Long-Term Automated Monitoring of Budgerigar Vocal Activity Using YAMNet

**DOI:** 10.3390/ani16203246

**Published:** 2026-10-15

**Authors:** Aldiyar Agishev, Irina Arifulova, Margulan Ibraimov, Nataliya Surovtseva, Serik Khokhlov, Dauren Zhexebay, Alisher Skabylov

**Affiliations:** 1 Faculty of Physics and Technology, Al-Farabi Kazakh National University, 71/23 Al-Farabi Ave., Almaty 050040, Kazakhstan; margulan.ibraimov@kaznu.edu.kz (M.I.); skhokh88@gmail.com (S.K.); zhexebay92@gmail.com (D.Z.); alisher.skabylov@kaznu.edu.kz (A.S.); 2 Laboratory of Comprehensive Earthquake Forecasting, National Center for Seismological Observations and Research, 75A Al-Farabi Ave., Almaty 050060, Kazakhstan; arif_irina@mail.ru (I.A.); natali.s71@mail.ru (N.S.)

**Keywords:** avian vocal activity, *Melopsittacus undulatus*, acoustic event detection, edge computing, YAMNet, vocal activity time series, seismo-biological monitoring

## Abstract

Continuous monitoring of animal behavior may provide quantitative information on responses to environmental changes; however, its application in seismobiological research requires reliable characterization of baseline behavioral variability. This study developed and evaluated an edge-based bioacoustic monitoring system for the continuous analysis of vocal activity in budgerigars (*Melopsittacus undulatus*). Ten birds were monitored under stable housing conditions from June 2025 to May 2026. Audio was processed locally on a Raspberry Pi-based system that combined a pretrained YAMNet feature extractor with a task-specific binary classifier. Vocal activity was quantified using the vocal activity second (VAS), defined as a 1 s interval containing at least one detected vocalization. Time-series analysis revealed a pronounced diel organization of vocal activity, with increased activity during daylight hours and minimal activity at night, together with seasonal variability. Wavelet and periodogram analyses identified a periodic component near 24 h, with weaker components within the 2–8 h range. These findings demonstrate the feasibility of autonomous edge-based monitoring of avian vocal behavior and establish a quantitative analysis of acoustic activity. The present study represents a methodological framework for the collection and analysis of avian acoustic signals using machine learning and computational signal processing approaches. The proposed approach provides a quantitative framework for investigating changes in avian behavior and for evaluating whether potential behavioral responses in vocal organization are associated with environmental factors.

## Simple Summary

Bird vocal activity changes naturally throughout the day and across seasons, and these normal patterns need to be understood before unusual changes in behavior can be identified. In this study, we developed an automated system for long-term monitoring of vocal activity in a group of budgerigars. The system continuously recorded sound and automatically detected bird vocalizations, allowing us to track vocal activity over one year without continuous manual observation. The results showed a clear daily pattern, with very low activity at night, a rapid increase in the morning, sustained activity during the daytime, and a decline in the evening. Vocal activity also varied across the year. The developed approach provides a practical way to establish normal patterns of bird vocal behavior and to identify periods that differ from these expected patterns. Such systems may be useful for long-term studies of animal behavior and for investigating how animals respond to changes in their environment.

## 1. Introduction

A retrospective analysis [1] found that behavioral responses in birds accounted for a substantial proportion of the documented cases of unusual animal behavior preceding environmental cataclysms. Vocalization is one of the most informative components of avian behavior. Birds communicate through acoustic signals whose characteristics, including frequency ranges, are often species-specific and vary with their communicative function and behavioral context.

Passive acoustic monitoring (PAM) is widely used to investigate avian vocalizations. Algorithms have been developed to identify bird species from their songs and calls for ecological monitoring purposes [2,3,4]. This approach relies on recording acoustic signals in natural environments to monitor avian activity with minimal human interference. Automated recording and analysis of vocalizations enable quantification of the temporal dynamics of acoustic activity, identification of diel and seasonal patterns, and long-term observations without the continuous presence of an observer.

Experimental studies indicate that avian behavioral and physiological responses depend on the source, characteristics, and biological relevance of an external stimulus. For example, avian responses to a natural stressor associated with predator presence have been examined using telemetry recordings of heart rate and locomotor activity [5]. Other studies have shown that avian responses may differ between exposure to natural acoustic conditions and different types of industrial noise [6]. These findings highlight the need to account for the nature of external stimuli when interpreting changes in animal behavior.

A previous study [7] proposed an Animal Sound Activity Detection (ASAD) system as a preliminary step toward leveraging unusual animal behavior to forecast emergency events. The proposed approach involved extracting acoustic features from animal sounds as mel-frequency cepstral coefficients (MFCCs), followed by classification with a multiclass support vector machine (SVM). The results demonstrated the feasibility of automatically detecting anomalous animal acoustic activity and highlighted the potential of machine learning methods for addressing such tasks.

A subsequent study [8] proposed an automated system for recognizing avian alarm calls to support the early detection of forest fires. Convolutional neural networks (CNNs) were used to classify the audio signals by automatically extracting discriminative features from spectrograms of the recordings. The results further demonstrated the potential of deep learning approaches for the automated analysis of bioacoustic signals.

Although both studies addressed distinct applied problems, they demonstrated that machine learning methods can automatically detect and classify bioacoustic signals. These findings provide a methodological basis for applying similar approaches to investigations of animal behavior as a potential indicator. However, applying such approaches, for example, in seismo-biological monitoring first requires characterization of the baseline temporal structure of animal acoustic behavior, including its natural variability [9,10]. Establishing this baseline is essential for quantitatively identifying deviations and subsequently examining their associations with external factors, including geophysical influences.

The aim of the present study is to develop and evaluate an automated system for the continuous recording, processing, and time-series analysis of avian acoustic activity, and to demonstrate its application for characterizing baseline temporal patterns of vocal behavior. This study is methodological in nature and is intended to establish a framework for long-term monitoring. The developed monitoring system and the resulting characterization of baseline activity are intended to support future studies assessing whether deviations in vocal activity may be associated with environmental factors.

In this study, we developed an automated edge-based system for real-time audio processing that uses the pre-trained YAMNet neural network [11] to generate audio embeddings and a task-specific classifier trained on a labeled dataset compiled for this study. This architecture enables the automatic detection of avian vocalizations and their conversion into acoustic activity time series, which can subsequently be analyzed to identify deviations in vocal activity. Whereas previous systems were developed for discrete, event-oriented applications, the present system is designed for long-term, autonomous passive bioacoustic monitoring and analysis of temporal patterns in avian behavior.

## 2. Materials and Methods

### 2.1. Study Species

Budgerigars (*Melopsittacus undulatus*) were selected as the study species because they are highly social birds that rely extensively on acoustic communication in a wide range of behavioral interactions. This species has a well-characterized and complex learned vocal repertoire, including contact calls produced by both males and females, as well as other vocalizations associated with different social and behavioral contexts [12,13].

This species was also selected because budgerigars can be housed for extended periods under controlled conditions, enabling continuous acoustic monitoring without substantially disrupting their usual activity patterns. These characteristics enabled the collection of long-term acoustic time series and the characterization of natural diel and seasonal variability in vocal activity. Their suitability for group housing, relative ease of care, and widespread availability further support their use as a model species for experimental bioacoustic research and the subsequent evaluation of biological monitoring systems at fixed monitoring sites.

### 2.2. Housing Conditions and Acoustic Monitoring Setup

The study was conducted from June 2025 to May 2026 at the biological research station of the National Scientific Center for Seismological Observations and Research, located within the grounds of the Main Botanical Garden in Almaty, Kazakhstan. Ten adult, sexually mature budgerigars (five males and five females) were housed together in an aviary measuring 1.0 × 1.8 × 2.0 m, situated in a separate 25 m^2^ room with ample natural light. The birds were fed once daily according to a fixed schedule. Routine bird-care procedures and room inspections were conducted during the birds’ normal daytime activity period and followed a stable daily schedule throughout the entire monitoring period.

Acoustic monitoring was conducted under conditions that approximated the birds’ routine housing environment as closely as possible. No major sources of mechanical or equipment-generated noise were present either in the aviary room or on the grounds of the biological station, while natural environmental sounds remained part of the ambient acoustic background.

This setup enabled long-term recording of avian vocal activity under relatively stable housing conditions and a controlled feeding schedule. Maintaining these conditions was essential for establishing a baseline time series of vocal activity against which diel, seasonal, and other variations in avian acoustic behavior could subsequently be assessed.

### 2.3. Quantification of Vocal Activity

One of the principal methodological challenges in long-term bioacoustic monitoring is converting continuous audio recordings into quantitative variables suitable for statistical analysis [14]. Several approaches are currently used for this purpose, including analysis of the spectrotemporal characteristics of individual vocalizations, counting acoustic events or automated detections within predefined time intervals, and calculating acoustic indices that describe the distribution of acoustic energy and soundscape complexity [15,16,17].

Acoustic indices can substantially reduce the dimensionality of large audio datasets. However, their values are influenced not only by the vocal activity of the target species but also by overall soundscape composition, background noise, and calculation parameters, which can hinder direct biological interpretation [18,19,20]. In single-species studies, one approach to quantifying vocal activity is to automatically detect vocalizations and aggregate the detections over predefined time intervals. Metrics derived in this way have been used to analyze diel and seasonal patterns in avian vocal activity [21,22].

For this study, we adopted a metric primarily designed for the long-term monitoring of a bird group. We defined a vocal activity second (VAS) as a 1 s interval containing at least one detected vocalization. Successive 1 s intervals were thus represented as binary observations of vocal presence or absence. These observations were summed within consecutive aggregation windows, converting the continuous audio stream into a numerical time series of vocal-active seconds per window. Unlike counts of individual calls, VAS does not require complex group vocalizations to be segmented into discrete events. This is particularly advantageous when vocalizations from multiple birds overlap or when prolonged group chatter occurs.

The resulting time series was used to characterize diel dynamics, descriptive statistical properties, and periodicity, and to evaluate potential deviations from baseline using time-series methods, including spectral and wavelet analyses. VAS therefore provides a quantitative representation of baseline vocal activity to support subsequent automated screening for candidate anomalous periods.

### 2.4. Audio Acquisition and Preprocessing

The study employed a portable audio recording and analysis system built around a Raspberry Pi 4 Model B single-board computer (Raspberry Pi Ltd., Cambridge, UK). The system provided continuous, real-time acquisition and on-device processing of budgerigar vocal activity. Audio was acquired through a ZOOM F3 USB audio interface (ZOOM Corporation, Tokyo, Japan) connected to an AKG C414 XLS multipattern condenser microphone in the wide-cardioid polar pattern (AKG Acoustics GmbH, Vienna, Austria) positioned 2 m from the aviary and oriented toward it. The complete external dimensions of the aviary fell within the full width at half maximum (FWHM) of the microphone’s directional sensitivity pattern. The input gain was adjusted during preliminary testing to prevent clipping and was subsequently held constant throughout the study. Audio was recorded in 32-bit WAV format at a sampling rate of 48 kHz, providing sufficient bandwidth for the target frequency range of 125–7500 Hz. The digitized audio stream was transferred to the computing module for real-time processing. A ring buffer supported continuous acquisition by allowing incoming audio to be recorded while previously acquired segments were processed without interrupting the data stream. The Raspberry Pi system clock was synchronized using Network Time Protocol (NTP). All acoustic records and derived VAS observations were stored with timestamps based on the synchronized system clock.

**Figure 1:**
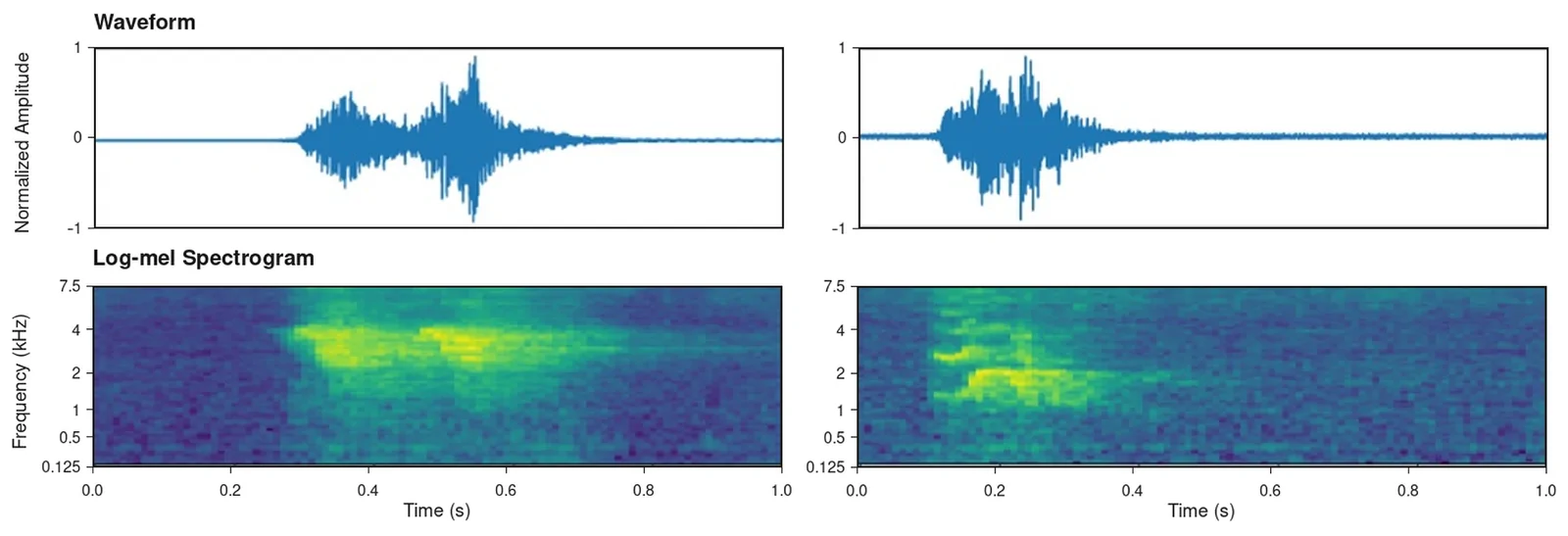
One-second audio waveform (**top**) and corresponding mel spectrogram (**bottom**) of a recorded budgerigar vocalization. In the mel spectrogram, color represents spectral magnitude, with darker blue tones indicating lower acoustic energy and brighter green tones indicating higher acoustic energy.

Before neural network inference, the audio was converted to mono, resampled to 16 kHz, and scaled to floating-point values ranging from −1 to +1 to meet the model’s input requirements. Within YAMNet, the input waveform was divided into overlapping 0.96 s frames with a hop size of 0.48 s, resulting in 50% overlap between adjacent frames. To calculate VAS, each non-overlapping 1 s interval was classified as vocal-active if at least one inference frame overlapping that interval was classified as containing a vocalization; otherwise, it was classified as inactive. Multiple positive frames overlapping the same 1 s interval contributed to a single VAS. All preprocessing and inference were performed locally on the device under Python v3.10.13 using TensorFlow v2.15.1, NumPy v1.26.4, pandas v2.1.4, SciPy v1.11.4, and Matplotlib v3.8.2., including the TensorFlow Lite framework, with the YAMNet v1 model. Figure 1 presents a representative 1 s waveform segment and its corresponding mel spectrogram.

### 2.5. Automated Quantification of Avian Vocal Activity Using YAMNet

As defined above, vocal activity was quantified using the vocal activity second (VAS), with one VAS corresponding to a non-overlapping 1 s interval containing at least one detected vocalization. A 1 s interval was assigned one VAS when at least one overlapping inference frame was classified as containing a bird vocalization; multiple positive frames within the same interval were not counted separately. This metric characterized baseline diel patterns of vocal activity, including periods with no detected vocalizations and peak-activity intervals, providing a quantitative group-level vocal activity profile.

The initial objective of automated processing was to distinguish budgerigar vocalizations from non-target sounds, including environmental background noise and anthropogenic sounds. This separation was required to construct a continuous time series representing specifically avian vocal activity. YAMNet v1 [11] was used as a feature extractor to generate high-level audio embeddings from the log-mel spectrogram representation of each input frame.

The convolutional backbone of YAMNet is based on the MobileNetV1 architecture [23], which uses depthwise separable convolutions, comprising successive depthwise and pointwise operations instead of conventional convolutions in most layers. This design substantially reduces computational complexity and the number of model parameters. YAMNet was pre-trained on the AudioSet dataset [24] to recognize 521 acoustic event classes. In the present study, YAMNet was not used as a stand-alone sound classifier but as a high-level feature extractor for a task-specific downstream classification problem.

For each input waveform, YAMNet returned three outputs: frame-level class scores, a log-mel spectrogram, and a 1024-dimensional embedding vector. The embeddings provide a compact, high-level representation of the audio signal’s spectrotemporal characteristics and are obtained from the network layer preceding the final classification layer.

The standard YAMNet class scores were not used as the final classification output because the broad AudioSet categories do not fully capture the species-specific vocalizations of the study birds or the acoustic conditions at the monitoring site. Instead, the 1024-dimensional embeddings were passed to a task-specific binary classifier trained to distinguish “bird vocalization” from “non-target sound.” This approach represents a form of transfer learning in which representations learned from a large general-purpose dataset are used to develop a specialized classifier from a comparatively limited amount of labeled target-domain data.

The downstream classifier was trained on a custom audio dataset collected at the biological station between October 2024 and April 2025, prior to the principal monitoring period. All training recordings were acquired using the same Raspberry Pi-based recording unit, USB audio interface, microphone, microphone placement, gain setting, and recording parameters as those used during operational monitoring. Audio was stored in 32-bit WAV format at a sampling rate of 48 kHz. The total duration of the raw recordings was 240 h. The recordings were manually annotated to identify intervals containing budgerigar vocalizations. Intervals containing no target vocalizations were assigned to the non-target class and included ambient background noise, human speech, equipment-generated sounds, and other extraneous acoustic events. Using the same acquisition chain for model development and operational monitoring minimized device-related domain differences and enabled the classifier to adapt to the actual acoustic conditions at the study site.

The combined use of transfer learning and on-device inference enabled continuous, automated, real-time bioacoustic monitoring with low computational overhead.

### 2.6. Classifier Architecture, Dataset Partitioning, and Evaluation

The task-specific binary classifier operated on the 1024-dimensional embeddings generated for each audio frame by the pretrained YAMNet feature extractor. YAMNet remained frozen throughout classifier training. Before model fitting, each embedding dimension was standardized using the mean and standard deviation estimated exclusively from the training set. The same fixed standardization parameters were subsequently applied to the validation and independent test sets. The classifier architecture and training configuration are summarized in Table A1.

To minimize information leakage due to temporal overlap and the similarity between adjacent audio frames, data partitioning was performed both before frame selection and at the recording-group level. All frames from a continuous recording session, together with all sessions obtained on the same calendar day, were assigned exclusively to one subset. Recording groups were allocated to the training, validation, and independent test subsets in proportions of 70%, 15%, and 15%, respectively, while retaining representation across months and times of day.

Following group-level partitioning, a balanced dataset of 40,000 labeled frames was selected without changing the group assignments. The resulting class distribution is presented in Table A2.

**Figure 2:**
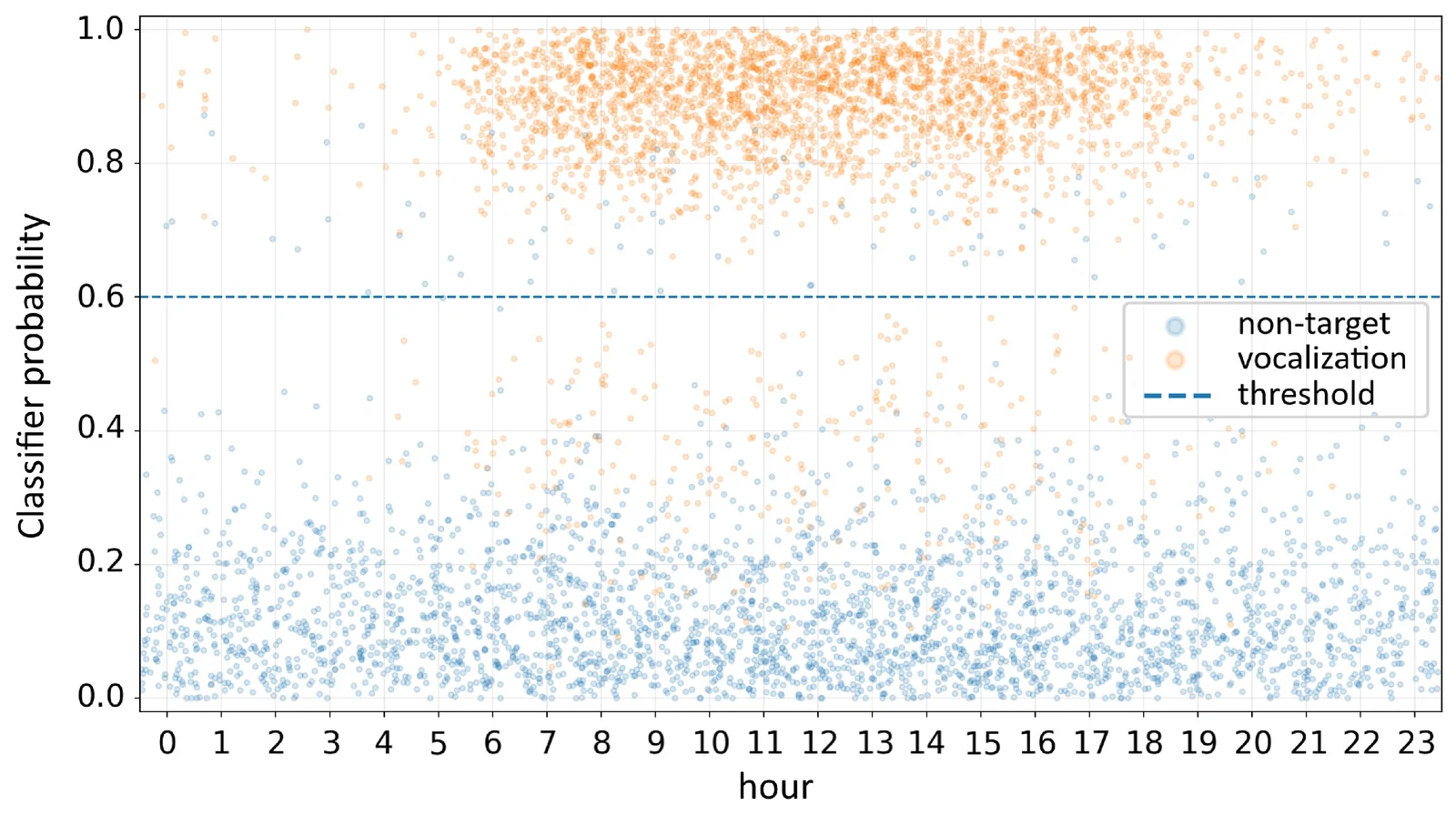
Classifier-predicted probabilities for manually labeled vocalization and non-target frames across the 24 h cycle in the independent test set. The corresponding confusion matrix is provided in Table A3.

The classifier output was interpreted as a budgerigar vocalization when the predicted probability was ≥0.60. This decision threshold was fixed before evaluation on the independent test set. The confusion matrix obtained for the independent test set at the fixed decision threshold of 0.60 is presented in Table A3 and Figure 2. Of the 6000 test frames, 5658 were classified correctly. The performance metrics derived from the confusion matrix and the continuous classifier scores are summarized in Table A4.

At the selected threshold, the classifier favored precision and specificity over sensitivity, with false-negative frames occurring more frequently than false-positive frames. The present analysis did not further categorize misclassified frames according to their acoustic characteristics.

## 3. Results

As part of this study, an automated system for recording and analyzing avian acoustic activity was developed and evaluated under long-term monitoring conditions. The system continuously acquired audio signals, automatically detected bird vocalizations within the continuous acoustic stream, and converted the detection outputs into quantitative time series. The analysis pipeline quantified the duration of vocal activity, as well as the dominant frequency and signal level of the detected events, thereby enabling tracking of temporal changes in these variables. The resulting data were used to characterize the baseline acoustic behavior of budgerigars housed at the biological research station of the National Scientific Center for Seismological Observations and Research, located in Almaty, Kazakhstan, and to identify variables that may be suitable for subsequent exploratory screening for candidate deviations from baseline.

For the initial temporal analysis, each non-overlapping 1 s interval was assigned a binary vocal-activity label, and positive intervals were summed within consecutive time bins to obtain the periodic vocal activity. The hourly values were then organized by calendar date and hour of day to generate an activity heatmap. Averaging the corresponding hourly values across observation days yielded the mean diel activity profile.

**Figure 3:**
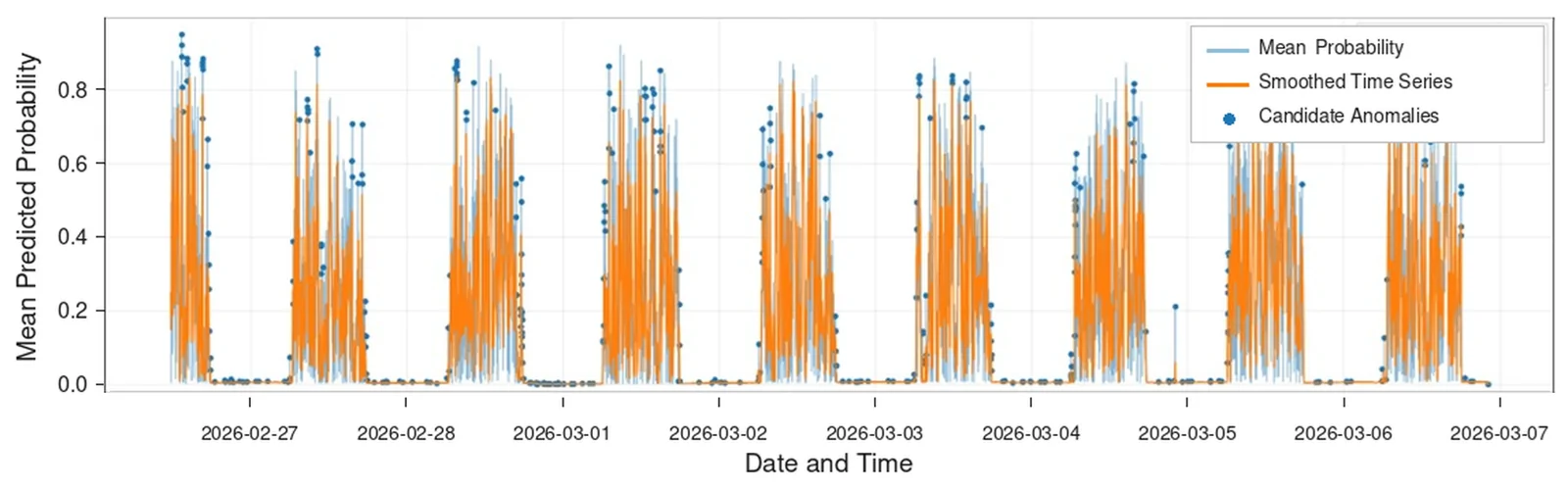
Temporal variation in the mean classifier-predicted probability of budgerigar vocalization from 26 February to 6 March 2026. Mean probabilities were calculated across the inference windows within consecutive 1 h intervals. The light-blue line shows the hourly mean predicted probability, the orange line represents the smoothed time series, and the blue markers indicate intervals flagged as candidate anomalies, represented by deviations greater than one standard deviation. Values range from 0 to 1, with higher values indicating a higher mean probability assigned to the budgerigar-vocalization class.

The classifier-derived time series, expressed as the mean predicted probability of budgerigar vocalization for each hourly interval, showed recurrent daytime elevations and values near zero at night, along with occasional high-amplitude peaks flagged as candidate deviations from the prevailing pattern (Figure 3). At this stage, a candidate anomaly was defined as an hourly observation deviating by more than one standard deviation from the overall distribution of classifier-derived activity observed during the monitoring period, without stratification by month. During the representative period from 26 February to 6 March 2026, the mean predicted probability increased after daylight onset, remained relatively high during the day, and declined sharply in the evening. Nevertheless, both the magnitude and within-day distribution of the classifier-derived scores varied among days, indicating natural day-to-day variability. Consequently, potential anomalies should not be identified solely from absolute increases or decreases in classifier output or hourly VAS. Changes in the temporal organization of acoustic behavior should also be considered, including shifts in the timing of active periods, changes in the amplitude of the diel cycle, and reductions in its regularity.

Elevated classifier scores occurred predominantly during daylight hours and decreased substantially at night. The recurrence of this day–night pattern across the analyzed interval is consistent with a diel organization of budgerigar vocal behavior. The concentration of candidate anomalies during nighttime and day–night transition periods demonstrates the limitation of applying a single global deviation criterion to a strongly time-dependent behavioral signal and motivates the subsequent introduction of a time-dependent baseline. The probability-based time series was used to visualize temporal changes in classifier output, whereas quantitative estimates of vocalization duration were based on thresholded VAS values.

**Figure 4:**
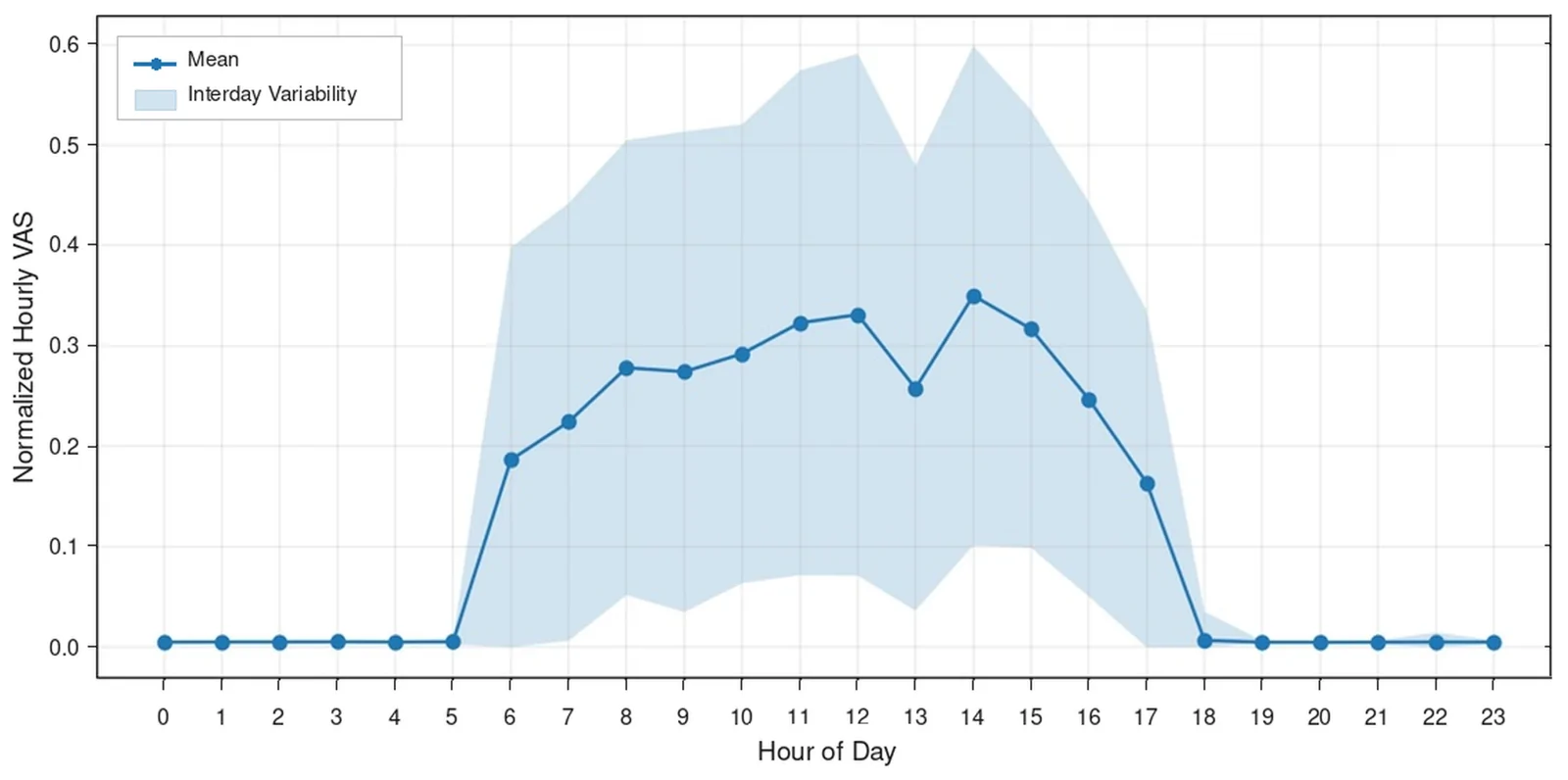
Mean diel profile of hourly budgerigar vocal activity. The points and solid line represent mean hourly VAS values calculated across all observation days, whereas the shaded band represents interday variability.

The mean diel profile, obtained by averaging the classifier-predicted probabilities for each hour across all observation days, revealed a characteristic daily pattern. The mean predicted probability increased markedly after dawn, reached its highest values during daylight hours, and subsequently decreased toward evening (Figure 4). The spread around the mean reflects the natural interday variability.

**Figure 5:**
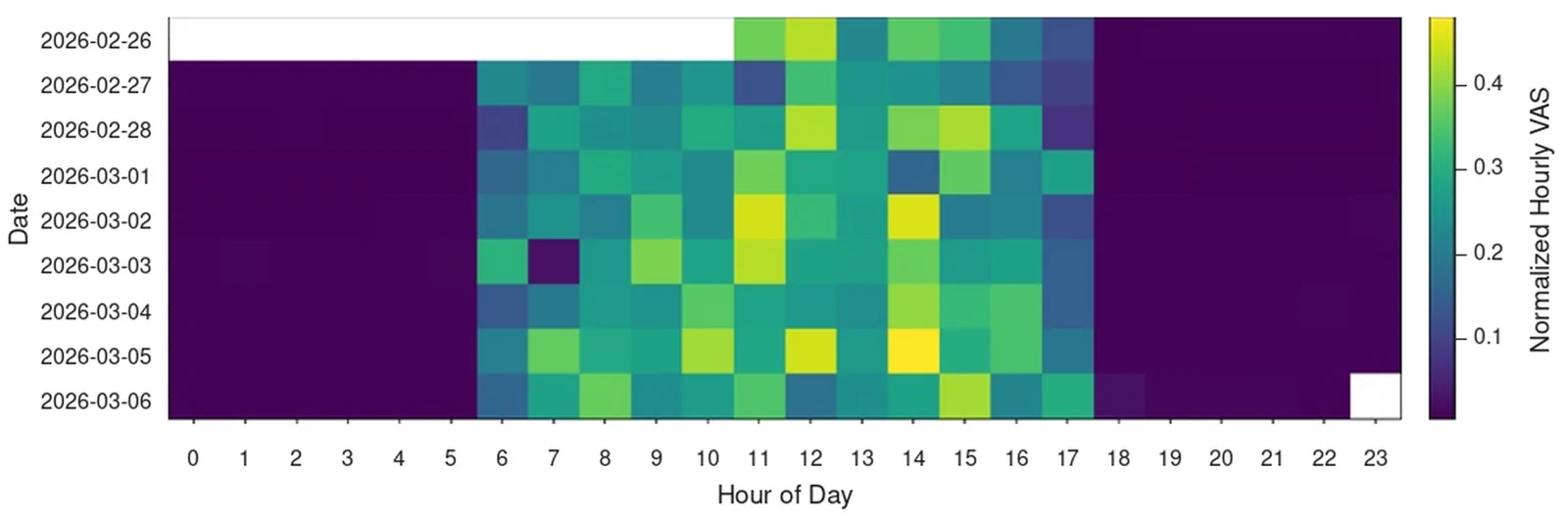
Date-by-hour heatmap of budgerigar vocal activity between 26 February and 6 March 2026. Each cell represents the hourly VAS value for the corresponding date and hour, with color intensity indicating the magnitude of vocal activity.

The date-by-hour heatmap visualized day-to-day variation in the classifier-derived probability of budgerigar vocalization and showed that higher probabilities were consistently concentrated during daylight hours (Figure 5). This representation provides a quantitative description of the temporal organization of detected vocal activity and can be used in subsequent analyses of longer-term seasonal patterns and potential deviations associated with external influences.

**Figure 6:**
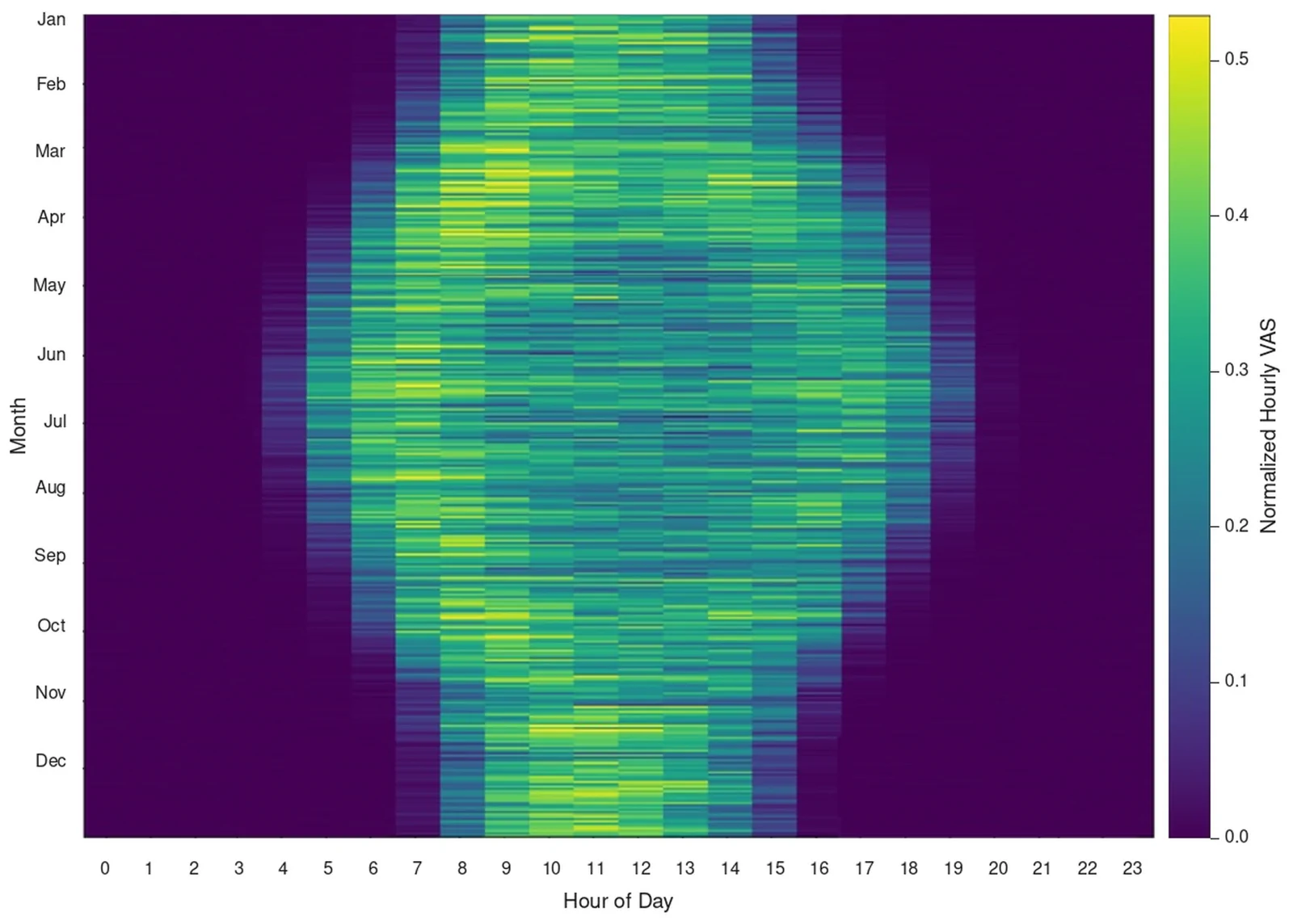
Annual date-by-hour heatmap of budgerigar vocal activity. Each row corresponds to an observation day, and each column represents an hour of the day. Color intensity indicates the corresponding hourly VAS value, with calendar months shown along the vertical axis.

To account for longer-term changes, the temporal organization of classifier-derived vocal activity was also examined over an annual timescale. The date-by-hour heatmap showed that both the timing and duration of the daytime activity window varied throughout the year (Figure 6). Aggregation over longer time intervals enabled estimation of the characteristic activity level and diel structure across different periods of the year. Incorporating this natural within-year variability into the baseline characterization reduces the likelihood that expected temporal changes will be misidentified as anomalous behavior.

Analysis of the annual time series indicated a predominantly diurnal pattern of budgerigar vocal activity, with the highest classifier-derived values generally occurring from the morning to early afternoon. An increase in the overall vocal-activity level was apparent in March and April. During summer, vocal activity remained relatively high but was distributed more evenly across daylight hours, resulting in a less pronounced daily peak. Thus, activity was less concentrated within a narrow time window than during the spring increase. In autumn, the overall activity level showed an apparent gradual decline. Nevertheless, the diel pattern remained broadly preserved, with the highest activity consistently occurring during daylight hours.

For biomonitoring purposes, vocal activity should therefore be compared with season- and time-of-day-specific baselines, because expected activity levels vary substantially across both temporal scales. The results indicate that the baseline acoustic activity of budgerigars cannot be adequately represented by a single constant level of vocalization. Rather, it constitutes a time-varying process with pronounced diel and day-to-day structure. Accordingly, automated screening for candidate deviations should use baseline values conditioned on the time of day and longer-term temporal variation. This approach helps distinguish candidate anomalies from ordinary changes in vocal behavior that form part of the natural variability of the monitored group.

To provide a simple quantitative representation of the time-dependent baseline, we summarized normalized VAS separately. For each hourly interval, the mean ($\mu_{h}$) and standard deviation ($\sigma_{h}$) were calculated across the monitoring period. To account for seasonal variation, the same statistics were additionally calculated separately for each month and hour of the day, yielding $\mu_{m,h}$ and $\sigma_{m,h}$ respectively, and resulting in a month-by-hour empirical baseline. For each observation, a standardized deviation score was calculated as:

(1) $$Z_{m,h}=\frac{{\mathrm{VAS}}_{m,h}-\mu_{m,h}}{\sigma_{m,h}}$$

where (${\mathrm{VAS}}_{m,h}$) is the observed normalized VAS at hour (*h*) in month (*m*). Observations with $|Z_{m,h}|>1$ were considered candidate deviations from the expected baseline. To reduce sensitivity to isolated fluctuations, we considered deviations exceeding this threshold for at least two consecutive hourly intervals.

The month-by-hour baseline therefore provided a simple quantitative framework for distinguishing expected temporal variability from unusually large deviations. Standardized deviation scores could subsequently be used to identify observations exceeding the normal range expected for a given month and time of day.

**Figure 7:**
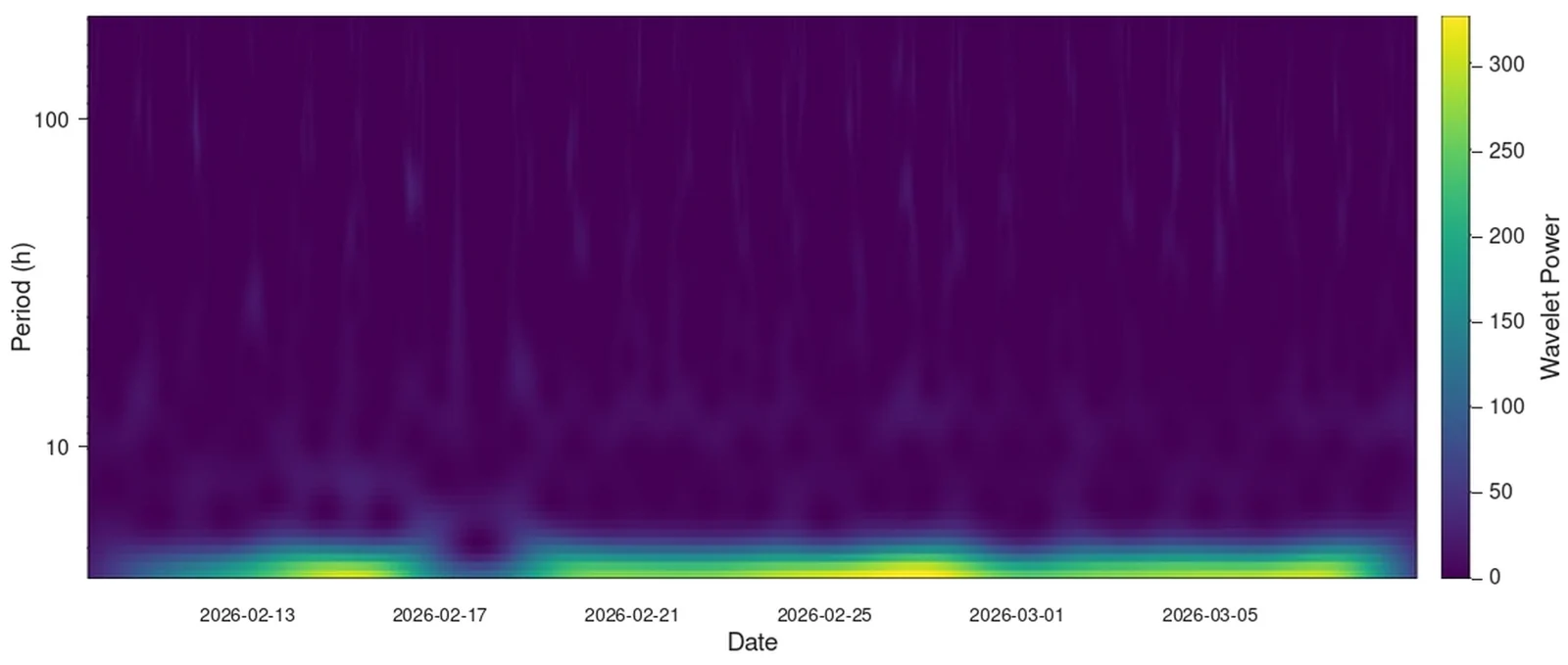
Continuous wavelet power spectrum of the hourly budgerigar VAS time series calculated using the complex Morlet wavelet. Time is shown on the horizontal axis, oscillation period (h) on the vertical axis, and color indicates wavelet power.

To characterize the periodic organization of budgerigar vocal activity and identify time-dependent changes in its periodic structure, a continuous wavelet transform (CWT) based on the complex Morlet wavelet was applied to the hourly VAS time series (Figure 7). Unlike the Fourier transform, which provides a global representation of the frequency content of the entire time series, wavelet analysis resolves both the characteristic oscillation periods and the time intervals during which the corresponding periodic components are most pronounced. This time-localized approach is particularly appropriate for analyzing animal behavior because the magnitude and temporal organization of vocal activity may vary over time in response to diel biological rhythms and external influences.

In the analyzed hourly VAS time series, the wavelet spectrum exhibited a prominent band of elevated power at periods of approximately 18–30 h, encompassing the 24 h periodicity. This component was present throughout a substantial proportion of the analyzed interval and was interpreted as reflecting the day–night organization of budgerigar vocal activity. Its occurrence was consistent with the mean diel profile shown in Figure 4, which demonstrated a marked increase in vocal activity during daylight hours and a decrease to near-minimal levels at night.

In addition to the 24 h component, lower-power features were observed at approximately 12 h periods. These features may reflect the within-day organization of vocal activity or represent the first harmonic of a non-sinusoidal 24 h cycle. However, the power and temporal persistence of individual periodic components varied over the analyzed interval. Thus, although the overall diel organization remained evident, its detailed rhythmic structure was time dependent. This finding is consistent with the nonstationary nature of avian vocal activity and supports the use of time–frequency analysis to characterize it.

**Figure 8:**
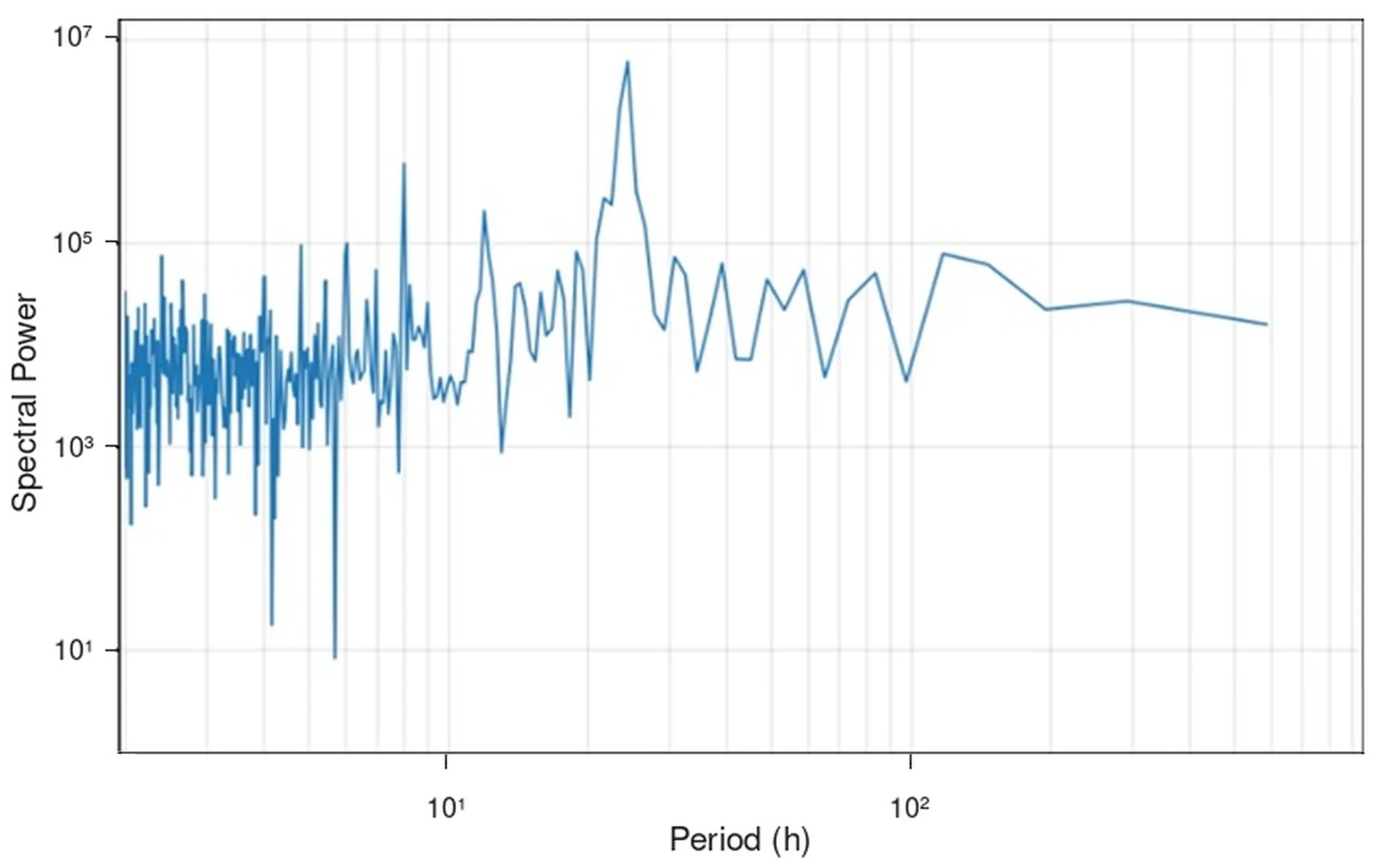
Periodogram of the hourly budgerigar VAS time series. Period and spectral power are plotted on logarithmic axes to display components spanning different timescales and power levels.

An additional assessment of the periodic structure of the hourly VAS time series was performed using a periodogram (Figure 8). Unlike the time-localized wavelet spectrum, which captures temporal changes in periodic components, the periodogram summarizes the distribution of spectral power across periods over the entire analyzed time series. The periodogram also showed a dominant peak at approximately 24 h, supporting the presence of this rhythm in budgerigar vocal activity. Lower-amplitude components were also observed at approximately 12 h and within the shorter-period range of 2–8 h. These features may reflect within-day variation in vocal activity or harmonics of the dominant non-sinusoidal 24 h cycle. Overall, spectral components at multiple timescales indicate that the temporal structure of vocal activity includes shorter-term fluctuations within the day.

Combining periodogram and wavelet analyses enabled identification of persistent timescales in vocal activity and assessment of time-dependent changes in the strength of the corresponding periodic components.

Candidate quantitative features for screening potential anomalies in avian vocal activity include mean hourly VAS, standardized deviations from a baseline adjusted for diel and seasonal variability, the coefficient of variation, spectral power within the 2–8 h and 18–30 h period bands, and increases in nighttime vocal activity. Collectively, these metrics enable quantification of increases in short-period spectral power, reductions in the persistence of the diel component, changes in the temporal organization of vocal activity, the duration of candidate anomalous episodes, and the magnitude and timing of deviations from baseline. Such changes may serve as candidate indicators of anomalous vocal behavior. However, their potential relevance as environmentally related characteristics must be evaluated separately by comparison with independent data.

## 4. Discussion

The present study is based on long-term monitoring of a single captive group of budgerigars. This design aligns with the study’s methodological objective: to develop and evaluate an approach for continuous acoustic recording, automated data processing, and time-series analysis, rather than to establish population- or species-level patterns of vocal activity. Therefore, the acoustic-activity characteristics reported here are specific to the monitored group and observation period and should be interpreted within this experimental context. These baseline patterns should not be directly extrapolated to other captive groups or to wild populations, where social structure, environmental conditions, resource availability, and other ecological factors may substantially influence vocal behavior.

The organization of acoustic activity identified in this study is fundamental to constructing an appropriate baseline model of vocal behavior. Changes in absolute hourly VAS values cannot, by themselves, be considered sufficient evidence of an anomalous state because vocal activity varies predictably throughout the day. Potential deviations should therefore be evaluated relative to a time-of-day-specific baseline and with reference to whether the normal temporal organization of acoustic behavior is preserved or disrupted.

Analysis of the annual time series also revealed seasonal variation in vocal activity: the increase in group-level VAS observed in March and April was followed by a gradual decline in activity toward autumn. The spring increase may have been partly related to reproductive activity. However, budgerigars do not have a single fixed breeding season across their entire geographic range, and the timing of reproductive activity varies depending on geographical and ecological conditions [25]. Thus, reproductive activity may have contributed to the spring increase, but the overall pattern observed is more appropriately interpreted as seasonal variation in baseline vocal activity.

Baseline behavior should be characterized using time-dependent quantitative features and spectral power within the short-period (2–8 h) and diel-scale (18–30 h) bands rather than by a single average activity level.

Feeding and standard husbandry procedures were conducted only during the birds’ daytime activity period and followed a consistent daily schedule throughout the entire monitoring period. These procedures therefore formed part of the stable background conditions under which the baseline vocal-activity pattern was established. Although they were not temporally associated with the onset or cessation of daily vocal activity, their potential contribution to within-day variation in activity cannot be completely excluded.

In the present framework, a candidate anomaly therefore represents a deviation from the established behavioral baseline rather than evidence of any specific external cause. Thus, false-positive control should be regarded primarily as a methodological problem of distinguishing genuine departures from expected vocal-activity patterns from normal temporal variability and external influences.

In long-term bioacoustic monitoring, candidate anomalies may arise not only from genuine departures from the established behavioral baseline but also from expected variability and external confounding influences. To reduce the risk of such false-positive flags, candidate anomalies should be evaluated relative to a time-dependent baseline rather than by applying a single global threshold. Recurrent or sustained deviations are likely to be more informative than isolated excursions, particularly when interpreted together with relevant environmental and husbandry covariates. Validation over longer observation periods and, where possible, across independent monitoring sites may further help distinguish reproducible departures from baseline behavior from local or transient disturbances.

A robust assessment therefore requires a multisite framework that accounts for the site-specific baseline of each biological monitoring station and evaluates the temporal concordance of observations across a regional network. Practical deployment of such a network would require standardized recording parameters, reliable time synchronization across stations, and consistent data-processing procedures to ensure comparability and reproducibility of the resulting measurements. When interpreting candidate anomalies flagged at different stations, consider differences in local acoustic environments and in the monitored animal groups. Synchronization across the regional network would also enable an assessment of whether comparable bioacoustic deviations occur at multiple stations within a common pre-event time window.

The next stage of this research will investigate avian responses to controlled environmental stimuli. Of particular interest is the separate experimental evaluation of airborne low-frequency sound and infrasound, as well as substrate-borne vibrations, including micro-vibrations, with parameters within the ranges proposed for signals preceding seismic events. Unlike the present study, which focused exclusively on baseline behavior, subsequent experiments conducted under controlled and ethically approved conditions will enable the development of a reference dataset comprising vocal and other behavioral responses to defined stimuli. This dataset may facilitate the identification of bioacoustic features that are particularly sensitive to departures from baseline behavior.

The VAS metric provides a quantitative measure of the presence and duration of vocal activity but does not distinguish between individual call types or their functional context. Therefore, further development of the monitoring system may include automated classification of vocalization types, enabling analysis not only of the overall duration of vocal activity but also of changes in its composition.

Bird vocal activity should therefore be characterized in greater detail to distinguish vocalizations associated with different social and functional contexts and, where independently validated, different affective states. Incorporating these characteristics may enable a more comprehensive assessment of anomalous acoustic behavior, improve the specificity of anomaly screening and help characterize the nature of a flagged deviation.

An additional limitation concerns the manual annotation of the training dataset. Although the labeling procedure followed a predefined binary criterion distinguishing intervals containing budgerigar vocalizations from non-target sounds, the annotations were not independently repeated by multiple observers, and formal inter-annotator agreement was therefore not assessed. Consequently, some degree of subjective labeling variability cannot be excluded, and such uncertainty may propagate into the trained classification model. The binary formulation of the task was intended to reduce ambiguity compared with the classification of functional vocalization types; however, future dataset development should include independent annotation by multiple observers and formal assessment of agreement using measurements. This would provide a more rigorous estimate of label reliability and further improve the reproducibility of the classification pipeline.

Work is currently underway to establish an annotated reference library of avian bioacoustic responses to various controlled acoustic, vibrational, and infrasonic stimuli. Such a dataset would support the transition from detecting general changes in vocal activity to investigating whether the likely stimulus category can be automatically classified using machine-learning methods. This represents an important step toward developing an intelligent automated monitoring system.

## 5. Conclusions

The present study focused on baseline acoustic behavior under routine housing conditions and in the absence of deliberately imposed stressors. The data revealed characteristic hourly and monthly patterns in budgerigar vocal activity and enabled the construction of a site-specific baseline for the monitored group of *Melopsittacus undulatus* at the Bioseismic Laboratory of the Botanical Garden of Almaty. These findings provide a methodological framework and an empirical baseline for the subsequent identification of quantitative deviations from expected vocal behavior.

Converting automatically detected vocalizations into continuous quantitative VAS time series enabled the transition from the analysis of individual acoustic events to the characterization of the temporal organization of avian vocal behavior. The hourly VAS series exhibited a pronounced 24 h pattern, with increased vocal activity during daylight hours and minimal activity at night, together with longer-term variation across the monitoring period. Wavelet and periodogram analyses identified a dominant spectral component centered near 24 h within the 18–30 h period band, while weaker components near 12 h and within the 2–8 h range reflected shorter-timescale variability and possible harmonics of the cycle. These results demonstrate that baseline vocal activity is strongly time-dependent and should therefore be characterized using both temporal and spectral features rather than a single average activity level.

Collectively, the results provide a quantitative framework for identifying candidate anomalies as deviations from the expected temporal organization of vocal activity. Such deviations may include changes in overall VAS, atypical nighttime activity, reduced persistence of the diel component, or changes in the relative power of characteristic periodic components. Candidate anomalies should be interpreted relative to time-of-day- and season-dependent baseline variability and regarded as screening events rather than evidence of any specific external cause. The proposed framework therefore provides a methodological basis for long-term automated bioacoustic monitoring and for subsequent comparison of candidate behavioral deviations with independently measured environmental variables. 

## Data Availability

The original contributions presented in this study are included in the article. Further inquiries can be directed to the corresponding author.

## Abbreviations

ASAD	Animal Sound Activity Detection
CNN	Convolutional neural network
CWT	Continuous wavelet transform
FWHM	Full width at half maximum
MFCC	Mel-frequency cepstral coefficient
PAM	Passive acoustic monitoring
ReLU	Rectified linear unit
SVM	Support vector machine
USB	Universal Serial Bus
VAS	Vocal Activity Second
WAV	Waveform Audio File Format
YAMNet	Pre-trained audio event classifier based on MobileNetV1

## Appendix A

**Table A1:** Architecture and training configuration of the task-specific binary classifier.

Component or Setting	Configuration
Input	1024-dimensional YAMNet embedding
Feature standardization	Feature-wise standardization using the training-set mean and SD
Dense layer 1	128 units, ReLU activation, L2 coeff. $10^{-4}$
Dropout layer 1	Dropout rate = 0.30
Dense layer 2	32 units, ReLU activation, L2 coeff.$10^{-4}$
Dropout layer 2	Dropout rate = 0.20
Output layer	One sigmoid unit
Loss function	Binary cross-entropy
Optimizer	Adam
Initial learning rate	0.001
Batch size	256
Maximum number of epochs	100
Early stopping	Validation-loss monitoring; patience = 10 epochs; restoration of the best-performing weights

**Table A2:** Distribution of labeled frames across the training, validation, and independent test subsets.

Dataset Subset	Recording-Group Allocation	Budgerigar Vocalization	Non-Target Sound	Total
Training	70%	14,000	14,000	28,000
Validation	15%	3000	3000	6000
Independent test	15%	3000	3000	6000
Total	100%	20,000	20,000	40,000

**Table A3:** Confusion matrix for the independent test set at a decision threshold of 0.60.

Reference Label	Predicted Budgerigar Vocalization	Predicted Non-Target Sound	Total
Budgerigar vocalization	2742 (TP)	258 (FN)	3000
Non-target sound	84 (FP)	2916 (TN)	3000
Total	2826	3174	6000

Abbreviations: TP, true positive; FN, false negative; FP, false positive; TN, true negative.

**Table A4:** Performance of the classifier on the independent test set.

Metric	Value
Accuracy	94.30%
Precision	97.03%
Recall (sensitivity)	91.40%
Specificity	97.20%
F1-score	94.13%

## References

[1] Serazetdinova B. (2007). Seismobiology: Theory, Methods, and Practice of Earthquake Prediction.

[2] Cai J., He P., Yang Z., Li L., Zhao Q., Pan F. (2023). A deep feature fusion-based method for bird sound recognition and its interpretability analysis. Biodivers. Sci..

[3] Chen X., Zeng Z. (2023). Bird sound recognition based on adaptive frequency cepstral coefficient and improved support vector machine using a hunter-prey optimizer. Math. Biosci. Eng..

[4] Xie J., Zhu M. (2023). Acoustic Classification of Bird Species Using an Early Fusion of Deep Features. Birds.

[5] Bisson I.A., Butler L.K., Hayden T.J., Romero L.M., Wikelski M.C. (2009). No energetic cost of anthropogenic disturbance in a songbird. Proc. R. Soc. B Biol. Sci..

[6] Curry C.M., Des Brisay P.G., Rosa P., Koper N. (2018). Noise Source and Individual Physiology Mediate Effectiveness of Bird Songs Adjusted to Anthropogenic Noise. Sci. Rep..

[7] Astuti W., Aibinu A.M., Salami M.J.E., Akmelawati R., Muthalif A.G. Animal sound activity detection using multi-class support vector machines. Proceedings of the 2011 4th International Conference on Mechatronics (ICOM).

[8] Permana S.D.H., Saputra G., Arifitama B., Yaddarabullah, Caesarendra W., Rahim R. (2022). Classification of bird sounds as an early warning method of forest fires using Convolutional Neural Network (CNN) algorithm. J. King Saud Univ.-Comput. Inf. Sci..

[9] Mei J., Li L., Zhang W., Shi J., Zhao S., Yong F., Ge X., Tong W., Zhou X., Cui P. (2026). Patterns of Vocal Activity of the Chinese Bamboo Partridge Using BirdNET Analyzer. Animals.

[10] Kucuktopcu O., Masazade E., Ünsalan C., Varshney P.K. (2019). A real-time bird sound recognition system using a low-cost microcontroller. Appl. Acoust..

[11] Google Research (2020). YAMNet: Pre-Trained Audio Event Classification Model. https://tfhub.dev/google/yamnet/1.

[12] Osmanski M.S., Seki Y., Dooling R.J. (2021). Constraints on vocal production learning in budgerigars (*Melopsittacus undulatus*). Learn. Behav..

[13] Tu H.W., Osmanski M.S., Dooling R.J. (2011). Learned vocalizations in budgerigars (*Melopsittacus undulatus*): The Relatsh. Contact Calls Warble Song. J. Acoust. Soc. Am..

[14] Gibb R., Browning E., Glover-Kapfer P., Jones K.E. (2019). Emerging opportunities and challenges for passive acoustics in ecological assessment and monitoring. Methods Ecol. Evol..

[15] Pieretti N., Farina A., Morri D. (2011). A new methodology to infer the singing activity of an avian community: The Acoustic Complexity Index (ACI). Ecol. Indic..

[16] Sugai L.S.M., Silva T.S.F., Ribeiro J.W., Llusia D. (2019). Terrestrial Passive Acoustic Monitoring: Review and Perspectives. BioScience.

[17] Kahl S., Wood C.M., Eibl M., Klinck H. (2021). BirdNET: A deep learning solution for avian diversity monitoring. Ecol. Inform..

[18] Bateman J., Uzal A. (2022). The relationship between the Acoustic Complexity Index and avian species richness and diversity: A review. Bioacoustics.

[19] Alcocer I., Lima H., Sugai L.S.M., Llusia D. (2022). Acoustic indices as proxies for biodiversity: A meta-analysis. Biol. Rev..

[20] Bradfer-Lawrence T., Desjonqueres C., Eldridge A., Johnston A., Metcalf O. (2023). Using acoustic indices in ecology: Guidance on study design, analyses and interpretation. Methods Ecol. Evol..

[21] Wu S.H., Ko J.C.J., Lin R.S., Chang-Yang C.H., Chang H.W. (2024). Evaluating community-wide temporal sampling in passive acoustic monitoring: A comprehensive study of avian vocal patterns in subtropical montane forests. F1000Research.

[22] Singer D., Kamp J., Hondong H., Schuldt A., Hagge J. (2026). Diel and seasonal vocal activity patterns revealed by passive acoustic monitoring suggest expert recommendations for breeding bird surveys need adjustment. J. Ornithol..

[23] Howard A.G., Zhu M., Chen B., Kalenichenko D., Wang W., Weyand T., Andreetto M., Adam H. (2017). MobileNets: Efficient Convolutional Neural Networks for Mobile Vision Applications. arXiv.

[24] Gemmeke J.F., Ellis D.P.W., Freedman D., Jansen A., Lawrence W., Moore R.C., Plakal M., Ritter M. Audio Set: An ontology and human-labeled dataset for audio events. Proceedings of the 2017 IEEE International Conference on Acoustics, Speech and Signal Processing (ICASSP).

[25] Wyndham E. (1983). Movements and Breeding Seasons of the Budgerigar. Emu-Austral Ornithol..

